# Unanticipated Antigens: Translation Initiation at CUG with Leucine

**DOI:** 10.1371/journal.pbio.0020366

**Published:** 2004-10-26

**Authors:** Susan R Schwab, Jessica A Shugart, Tiffany Horng, Subramaniam Malarkannan, Nilabh Shastri

**Affiliations:** **1**Division of Immunology, Department of Molecular and Cell BiologyUniversity of California, Berkeley, CaliforniaUnited States of America

## Abstract

Major histocompatibility class I molecules display tens of thousands of peptides on the cell surface for immune surveillance by T cells. The peptide repertoire represents virtually all cellular translation products, and can thus reveal a foreign presence inside the cell. These peptides are derived from not only conventional but also cryptic translational reading frames, including some without conventional AUG codons. To define the mechanism that generates these cryptic peptides, we used T cells as probes to analyze the peptides generated in transfected cells. We found that when CUG acts as an alternate initiation codon, it can be decoded as leucine rather than the expected methionine residue. The leucine start does not depend on an internal ribosome entry site–like mRNA structure, and its efficiency is enhanced by the Kozak nucleotide context. Furthermore, ribosomes scan 5′ to 3′ specifically for the CUG initiation codon in a eukaryotic translation initiation factor 2–independent manner. Because eukaryotic translation initiation factor 2 is frequently targeted to inhibit protein synthesis, this novel translation mechanism allows stressed cells to display antigenic peptides. This initiation mechanism could also be used at non-AUG initiation codons often found in viral transcripts as well as in a growing list of cellular genes.

## Abstract 2

Proteins have been identified for which a unique translational machinery makes use of unconventional start codons

## Introduction

Immune surveillance by cytotoxic T cells (CTLs) is a key mechanism for detecting and eliminating abnormal cells. These include cells infected with viruses or bacteria, and those that have suffered tumorigenic transformations (Townsend and Bodmer 1989). The antigen receptors of CTLs probe the repertoire of peptide/major histocompatibility complex (MHC) class I complexes on the target cell surface. Target cells display peptides derived from virtually all cellular translation products; the presence of foreign peptides, such as those derived from viral proteins, can trigger a T cell response. Each cell presents tens of thousands of distinct peptides as potential ligands for the CTL antigen receptor (Rammensee et al. 1993; Engelhard 1994). Most peptides are represented at fewer than ten copies per cell, and by some estimates only three copies of the antigenic peptide are sufficient for target cell lysis (Purbhoo et al. 2004). CTLs are thus a very sensitive probe for the peptides displayed by MHC class I.

The antigen-presenting cells (APCs), which include almost all nucleated cells, are also very efficient in generating peptides for display by MHC class I (Pamer and Cresswell 1998; Princiotta et al. 2003). In fact, they are so efficient that the complex mixture of peptides on the cell surface includes some peptides that should in theory never have been translated in the first place (Shastri et al. 2002). These peptides, referred to as “cryptic,” are derived from the 5′ and 3′ “untranslated” regions of the RNA or from alternate translational reading frames. Cryptic peptides have been identified as targets for CTLs specific for tumors as well as virus-infected cells (Mayrand and Green 1998; Cardinaud et al. 2004). Several studies have shown that these peptides can arise in tumor cells and cultured cell lines despite the absence of conventional AUG codons (Malarkannan et al. 1995a; Dolstra et al. 1999; Malarkannan et al. 1999; Ronsin et al. 1999). Recently, using a transgenic approach we demonstrated that such peptides can also be expressed in a variety of normal cells and can elicit CTL responses (Schwab et al. 2003). Remarkably, a distinct translation mechanism appeared to be responsible for their generation, because it was capable of decoding the CUG initiation codon as leucine rather than the expected methionine residue.

How APCs generate peptides using non-AUG codons remains obscure. It is believed that cells express only one class of initiator tRNA, RNA_i_^Met^, which is specific for AUG and is always charged with the methionine residue (Peabody 1989; Rajbhandary and Chow 1995). 40S ribosomes are preloaded with RNA_i_^Met^ and other initiation factors even before they approach the mRNA to be translated. Initiation at non-AUG codons is therefore thought to be caused by “wobble” in the pairing of the non-AUG codon with the anticodon of the RNA_i_^Met^ (Peabody 1989). This mispairing results in incorporation of the methionine residue at the non-AUG initiation codon. How cells initiate translation with a nonmethionine residue is thus not explained by current translational theory. It is nevertheless important to understand this mechanism not only because antigenic peptides can arise from non-AUG initiated translation, but also because expression of a growing number of genes appears to be mediated by translation initiated at non-AUG codons.

In this study, we used T cells as probes to analyze the translation mechanism that allows the generation of CUG-initiated antigenic peptides and decodes CUG as the leucine rather than the methionine residue. We found some similarities, but also key differences, between the translation mechanisms mediating initiation at conventional AUG versus CUG codons.

## Results/Discussion

### Decoding of the CUG Initiation Codon as Leucine Does Not Depend upon the mRNA Sequence

We had previously shown both in transfected cell lines and in a transgenic mouse model that CUG can be decoded as leucine when it serves as the initiation codon for the (CTG)-TFNYRNL peptide (the initiation codon is in parentheses and the remaining amino acids are in single-letter code) (Malarkannan et al. 1999; Schwab et al. 2003). Although there are numerous examples of non-AUG initiation codons, this is to our knowledge the only known instance in which the identity of the first residue was investigated and found not to be methionine, with the exception of initiation directed by cricket paralysis virus-like internal ribosome entry sites (CPV-IRES). Normally the RNA_i_^Met^ occupies the P site of the ribosome, where translation initiation begins. In contrast, the CPV-IRES binds the ribosome and positions it precisely so that initiation begins in the A site, without requiring RNA_i_^Met^ (Jan and Sarnow 2002). In the cricket paralysis virus, initiation begins with alanine encoded by GCU, and in the Plautia stali intestine virus initiation begins with glutamine encoded by CAA (Sasaki and Nakashima 2000; Wilson et al. 2000). Because there was no obvious IRES-like sequence in the transgene we had used, and non-CUG leucine codons failed to direct efficient translation, we suspected that a different mechanism might account for initiation at the CUG codon (Schwab et al. 2003). Therefore we first asked whether the leucine start was dependent upon the mRNA sequence.

To address this question, we identified the first amino acid of CUG-initiated peptides that were presented by MHC class I molecules and thus detectable by appropriate T cells. In this assay, we cotransfected cells with cDNA constructs encoding the peptide as well as the MHC molecule that binds and presents the peptide on the cell surface. Binding to the MHC protects the initiation residue from being removed by cellular proteases, which can trim antigenic peptides in the cytoplasm or the endoplasmic reticulum (Kloetzel 2004; Rock et al. 2004). We then extracted the peptides translated in the cells and separated them by reverse-phase high performance liquid chromatography (RP-HPLC), using conditions in which the methionine-initiated peptide elutes at a different time from the leucine-initiated peptide. Finally, we assayed the HPLC fractions for the presence of the peptide. We added to each fraction a T cell hybridoma that cross-reacts with the methionine and the leucine-initiated forms of the peptide, as well as appropriate APCs that will display the peptide on their MHC molecules. We measured the T cell response to each fraction. By comparing the retention time of the T cell-stimulating fractions from the cell extract with the retention time of synthetic peptides, we can determine the identity of the peptide made by the cells. This assay has several advantages over conventional assays used to detect initiating amino acids: MHC molecules protect the first amino acid from cleavage, T cells can detect nonmethionine residues with high sensitivity, and we can analyze the products of translation initiation in vivo.

**Figure 1:**
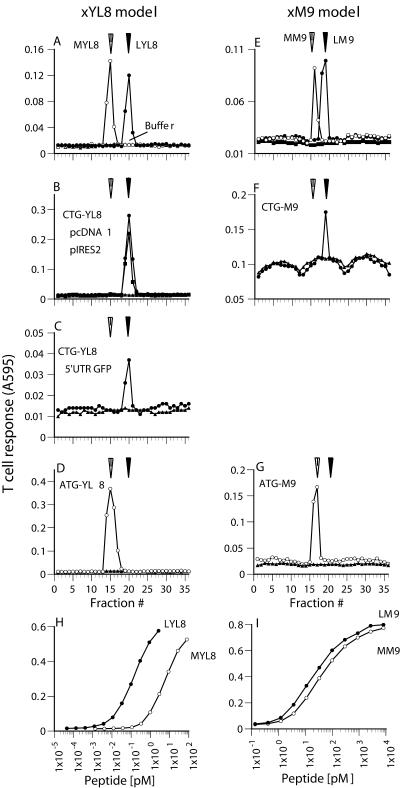
The CUG Initiation Codon Is Decoded as Leucine Independent of RNA Sequence (A) The indicated synthetic peptides mixed with extracts from untransfected COS-7 cells were separated by RP-HPLC. Each fraction was tested for BCZ103 T cell-stimulating activity with K^b+^B7.2^+^ L cells as APCs. After overnight incubation, the β-galactosidase induced in activated T cells was measured using the substrate chlorophenol red-β-pyranoside, which yields a colored product with absorbance at 595 nm. The arrows indicate the reproducible peak elution times for the MYL8 and the LYL8 peptides. Injections of buffer alone (Buffer) were carried out under identical conditions, and the fractions were assayed in parallel to ensure absence of cross-contamination between runs. (B–D) Extracts from COS-7 cells transfected with cDNA encoding K^b^ and the indicated constructs were separated by RP-HPLC. Fractions were tested for BCZ103 T cell-stimulating activity as in (A). (E) The indicated synthetic peptides mixed with extracts from untransfected COS-7 cells were separated by RP-HPLC. Each fraction was tested for the specific DBFZ T cell-stimulating activity with D^b+^B7.2^+^ L cells as APCs as in (A). The arrows indicate the reproducible peak elution times for the MM9 and the LM9 peptides. (F and G) Extracts from COS-7 cells transfected with cDNA encoding D^b^ and the indicated constructs were separated by RP-HPLC. Fractions were tested for DBFZ T cell-stimulating activity as in (E). (H) A range of concentrations of the indicated synthetic peptides was tested for BCZ103 T cell-stimulating activity with K^b+^B7.2^+^ L cells as APCs. (I) A range of concentrations of the indicated synthetic peptides was tested for DBFZ T cell-stimulating activity with D^b+^B7.2^+^ L cells as APCs.

To determine if decoding of the CUG initiation codon as leucine was influenced by 5′ or 3′ untranslated regions (UTRs) of the mRNA, we transfected COS-7 cells with cDNA encoding the K^b^ MHC molecule and the *(CTG)-TFNYRNL* peptide ([CTG]YL8). We refer to this as the xYL8 model. We placed the peptide in three different contexts: first, in the pcDNA1 vector; second, upstream of the IRES in the pIRES2-eGFP vector; and, finally, in the 5′ UTR of green fluorescent protein (GFP) in the pcDNA1 vector. The peptides translated in the transfected cells were extracted and fractionated by HPLC. Each fraction was tested for the presence of the leucine-initiated LTFNYRNL (LYL8) peptide and the methionine-initiated MTFNYRNL (MYL8) peptide using BCZ103 T cells and K^b^-expressing L cells (a fibroblast cell line) as APCs. With each construct we found a single peak of antigenic activity that eluted in the same fraction as the synthetic LYL8 peptide (Figure 1A–1C). In contrast, when the cells were transfected with a construct encoding the ATG-initiated peptide ([ATG]-TFNYRNL), the single peak of antigenic activity eluted in fractions identical to those containing the synthetic MYL8 peptide (Figure 1A and 1D). This result rules out the possibility that the apparent use of leucine was due to post-translational modification of the MYL8 peptide, which caused it to coelute with the LYL8 peptide. We conclude that the cells were capable of decoding the initiating CTG codon as leucine regardless of the 5′ or 3′ sequences flanking the LYL8 coding sequence.

We next tested whether the LYL8 coding sequence itself enabled the leucine start. We examined the initiating amino acid used for a different peptide presented by the D^b^ MHC class I molecule that satisfied the conditions required for our assay: that an MHC molecule present the peptide, that HPLC allow distinction between the leucine- and methionine-initiated forms, and that a T cell cross-react with the peptides with leucine or methionine residues at the first position. When COS-7 cells were transfected with cDNA constructs encoding the D^b^ MHC molecule and the *(CTG)-SNEN-METM peptide derived from the influenza nucleoprotein, only the leucine-initiated LM9 peptide was detected in the HPLC-fractionated cell extracts (Figure 1E and 1F). We again assessed whether the apparent use of leucine could be due to post-translational modification of the methionine-initiated peptide. We transfected cells with a construct containing the ATG initiation codon in place of the CTG codon. Analysis of cell extracts after HPLC fractionation now revealed a single peak of antigenic activity that eluted in the same fractions as the synthetic MM9 peptide (Figure 1E and 1G). Similar results were also obtained with the (CTG)-SHL8 peptide that was presented by the D^b^ MHC class I molecule (Malarkannan et al. 1995b) (unpublished data). We conclude that the peptide sequence itself was irrelevant to the decoding of the CTG initiation codon as leucine.

At present it is difficult to quantify the fraction of the total translated material that is initiated with leucine, because the different peptides may have different stabilities in the cell. Furthermore, the T cell hybridomas could respond to the methionine- and leucine-initiated peptides with differing sensitivities. Indeed, the BCZ103 T cell hybridoma responds to LYL8 approximately 30-fold better than to MYL8 (Figure 1H). In contrast, the DBFZ T cell hybridoma recognizes its leucine- or methionine-initiated cognate peptides with comparable sensitivity (Figure 1I). Nonetheless, translational initiation with the leucine residue is readily detected.

**Figure 2:**
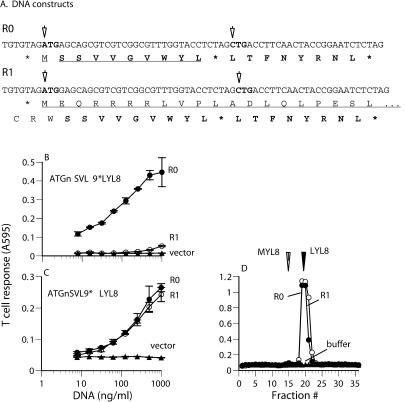
Expression of the Cryptic LYL8 Peptide Is Independent of the Efficiency of Upstream Translation Initiation Events (A) The nucleotide sequences of R0 and R1 constructs encode the SVL9 peptide followed by a termination codon and the LYL8 peptide. In the R0 construct, the SVL9 peptide is in frame with an ATG initiation codon. In the R1 construct, a single nucleotide is inserted after the ATG codon and causes the SVL9*LYL8 coding sequence to be out-of-frame with the ATG. The in-frame translation products are underlined and arrows indicate the potential initiation codons. (B and C) The R0 and R1 constructs were transfected into Lmtk^–^ cells along with the appropriate MHC molecule. They were tested for SVL9/D^b^ expression using the 30NX/B10Z hybridoma (B) and LYL8/K^b^ expression using the BCZ103 T cell hybridoma (C). (D) Extracts from COS-7 cells transfected with cDNA encoding K^b^ and the indicated constructs were separated by RP-HPLC. Fractions were tested for BCZ103 T cell stimulating activity as in Figure 1.

In previous studies, we explored the possibility that leucine was used as the first amino acid because the ribosome may have begun at an upstream alternate initiation codon (there are no upstream ATGs in these constructs) and read through the stop codon before the CTG initiation codon in the LYL8 coding sequence. We showed that increasing the number of stop codons upstream of CTG from one to six had no effect on LYL8 expression, and that substituting the CTG codon with other leucine-encoding triplets essentially ablated peptide expression (Malarkannan et al. 1999; Schwab et al. 2003). We assessed the possibility of translational read-through in the different model systems used here. If read-through were responsible for LYL8 expression, one would predict that expression of the downstream LYL8 would be proportional to expression of the upstream sequence. To test this hypothesis, we made DNA constructs in which the LYL8 coding sequence was placed directly after the stop codon terminating translation of another peptide, SSVVGVWYL (SVL9) (Mendoza et al. 1997). We then placed the [SVL9*LYL8] cassette in-frame (R0) with and out-of-frame (R1) with an ATG initiation codon (Figure 2A). These constructs were transfected into cells along with the appropriate MHC molecules, and peptide expression was assayed with the 30NX/B10Z T cell hybridoma specific for the SVL9/D^b^ complex (Figure 2B) and the BCZ103 T cell hybridoma specific for the LYL8/K^b^ complex (Figure 2C). T cells were added directly to the transfected cells without an intervening extraction step. Cells expressing the R0 cassette were highly active as APCs. Shifting the cassette out-of-frame with the initiating ATG in the R1 construct dramatically reduced expression of the SVL9 peptide. Remarkably, the cells transfected with either the R0 or the R1 constructs were equivalent in their ability to stimulate the LYL8-specific T cells (Figure 2C). To confirm that the CTG initiation codon was decoded as leucine, we analyzed HPLC fractionated cell extracts (Figure 2D). Again, a single peak of activity was found in the fractions that matched the leucine-initiated LYL8 synthetic peptide. Thus, translation of the LYL8 peptide was independent of upstream conventional translation initiation events, and translational read-through events were not detected.

We also considered the possibility that leucine was used as the first amino acid because of an RNA modification that introduced an AUG immediately upstream of the peptide-coding sequence. However, the experiments below show that the 5′ UTR influences the leucine start because it is affected by the Kozak context, by the presence of an upstream hairpin, and by the presence of upstream initiation codons. Together, these findings demonstrate that the mRNA remained intact.

### The Kozak Context Affects the Efficiency of Initiation at CUG

The efficiency of initiation at a given AUG codon depends on the identity of the surrounding nucleotides. These nucleotides, commonly referred to as the “Kozak context,” have a substantial influence on protein synthesis. Kozak found that GCCACC**AUG** G is optimal, and that the nucleotides at positions –3 and +4 are the most influential, while the nucleotide at the –6 position exerts a smaller effect. Changing these nucleotides can change protein expression by 20-fold (Kozak 1987a, 1987b). To determine whether the leucine start was affected by the nucleotides surrounding the CUG initiation codon, and to allow better prediction of probable CUG initiation codons, we varied the nucleotides at positions –6, –3, and +4 relative to the CUG codon.

**Figure 3:**
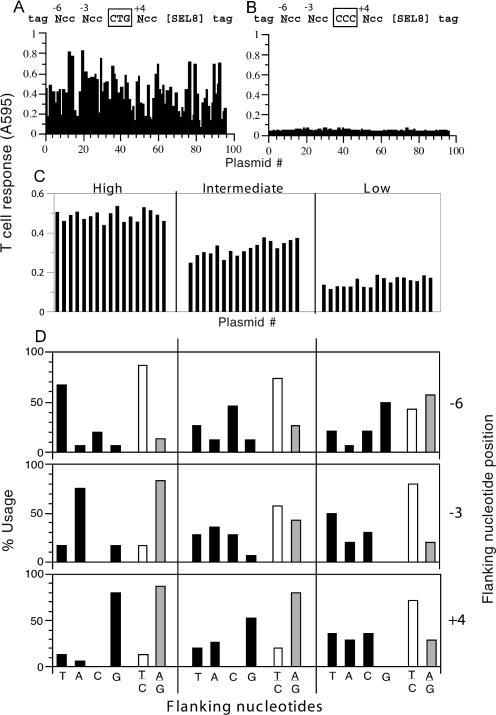
The Optimal Nucleotide Context for the CUG Initiation Codon (A and B) The indicated degenerate oligonucleotides were cloned into the pcDNA1 vector. “N” represents any one of T, A, C, and G nucleotides. The CTG or CCC initiation codons are boxed and the peptide coding sequence is indicated by [SEL8]. 96 randomly picked plasmids for the CTG-initiated peptide and an equal number for the CCC-initiated peptide were purified. The plasmids were transfected into COS-7 cells along with the K^b^ MHC class I molecule, and the T cell response was measured. Each bar represents the T cell response to cells transfected with an individual plasmid. (C) Three sets of 18 representative plasmids, each yielding high, intermediate, and low responses (as shown) were selected for nucleotide sequencing. (D) Summary of the nucleotide sequences of plasmids yielding high, intermediate, and low responses. The left, middle, and right panels, respectively, correspond to the plasmids shown in (C). Each panel shows the percent of each nucleotide found at the –6, –3, and +4 degenerate positions indicated by the “N” in A. For example, the upper left square shows that, of the high T cell-stimulating plasmids, the –6 position was T for 67%, A for 6.7%, C for 20%, G for 6.7%, a pyrimidine (T or C) for 87%, and a purine (A or G) for 13%.

We inserted synthetic oligonucleotides *( NCCNCC**CTG** NCC)SEL8* into the pcDNAI vector, where N represents the degenerate nucleotides flanking the CTG initiation codon for the SLVELTSL (SEL8) peptide presented by the K^b^ MHC molecule to bm1BZ19.4 T cells (Malarkannan et al. 1996). We transfected COS-7 cells with plasmid DNA from 96 individual randomly picked bacterial colonies together with the cDNA for K^b^. When the transfected cells were tested for their ability to stimulate bm1BZ19.4 T cells, we noticed a substantial variation in the T cell response, suggesting that the plasmids differed in their ability to express the SEL8 peptide (Figure 3A). As a negative control, none of the cells transfected with each of the 96 plasmid DNAs encoding the minigene *( NCCNCC**CCC** NCC)SEL8* stimulated the T cells, demonstrating that initiation activity was restricted to the CTG codon (Figure 3B). To identify which nucleotides were associated with the variation in peptide expression, we determined the nucleotide sequences of three sets of 18 CUG-initiated constructs that yielded high, intermediate, or low T cell responses (Figure 3C). As summarized in the panels in Figure 3D, among the plasmids that yielded high responses, 70% had T at position –6, 75% had A at position –3, and 80% had G at +4. Conversely, among the plasmids that yielded low responses, these nucleotides were infrequent. Thus, the optimal context for the CUG mediated initiation is TCCACC**CUG** G, which is in close agreement with Kozak's consensus sequence ( GCCACC**AUG** G) for the AUG start and extends earlier findings that showed that an A at +5 and U at +6 can also enhance initiation at the CUG codon (Boeck and Kolakofsky 1994; Grunert and Jackson 1994). Furthermore, the fact that CUG initiation activity was influenced by the Kozak context provides additional evidence that the CUG codon was decoded during translation initiation rather than translation elongation.

### An Excellent Kozak Context Enhances Both the CUG/Leucine and the CUG/Methionine Starts

In the above model, because the initiation codon was not included within the final SEL8 antigenic peptide product protected by the MHC molecule, the identity of the amino acid residue specified by the CUG initiation codon could not be determined. Thus, we could not distinguish whether the Kozak context affected the leucine start, the methionine start, or both. To resolve this question, we turned to the (CTG)YL8 model, in which the predominant T cell-stimulating activity is the leucine-initiated LYL8 peptide (see Figure 1A–1C). At the –6 and –3 positions, we placed the best (T, A) and the worst (G, T) nucleotides. We were unable to vary the +4 position from the original A, because that would alter the peptide's second amino acid and likely affect its detectability by the BCZ103 T cell hybridoma.

**Figure 4:**
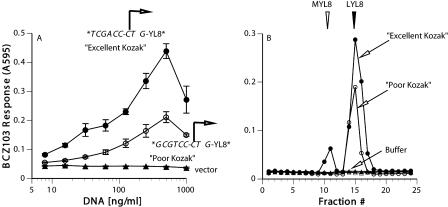
The Kozak Context Enhances the Leucine as well as the “Wobble” Methionine Start (A) Lmtk^–^ cells were transfected with K^b^ cDNA and the indicated “Excellent” and “Poor” constructs encoding the (CTG)YL8 peptide. They were tested for LYL8/K^b^ expression using the BCZ103 T cell hybridoma. (B) Extracts from COS-7 cells transfected with cDNA encoding K^b^ and the indicated constructs were separated by RP-HPLC. Fractions were tested for BCZ103 T cell-stimulating activity with K^b+^B7.2^+^ L cells as APCs. The arrows indicate the peak elution positions for the MYL8 and the LYL8 peptides. T cell responses to fractions collected after injecting sample buffer alone (Buffer) are also shown to indicate absence of cross-contamination between runs.

We first transfected cells with the two constructs as well as the appropriate K^b^ MHC cDNA. After 2 d the transfected cells were assayed for their ability to stimulate the BCZ103 T cell. Cells expressing the LYL8 peptide with its CTG initiation codon in the “Excellent Kozak” context (T at –6, A at –3) were superior to those with the CTG codon in a “Poor Kozak” context (G at –6, T at –3) in stimulating the T cell response (Figure 4A). Next, we extracted peptides from the cells expressing the two constructs and analyzed the antigenic activities after HPLC fractionation. Again, a single peak of activity corresponding to the leucine start was detected in the extract of cells expressing the “Poor Kozak” construct (Figure 4B). In contrast, in the extract from cells expressing the “Excellent Kozak” construct not only was the total amount of the LYL8 peptide higher, but a new activity peak corresponding to the methionine-initiated MYL8 peptide was also clearly detected. By comparing the T cell response to the cell extracts with the response to known quantities of synthetic peptides, we determined that the construct with the CTG initiation codon in an “Excellent Kozak” context yielded approximately 6-fold more LYL8 peptide than the construct with CTG in a poor context. The amount of MYL8 peptide increased as well, but the change cannot be quantified, because MYL8 was undetectable in extracts of cells transfected with the construct containing the CTG initiation codon in the “Poor Kozak” context (Figure 4B). We conclude that the Kozak context enhanced not only the overall efficiency of the CTG/leucine start, but also the ability of the CTG codon to be decoded in the conventional “wobble” mode as the methionine residue.

### Ribosomes Scan 5′ to 3′ for the CUG Initiation Codon

In most cases, ribosomes bind mRNA at the 5′ cap and scan in the 3′ direction for the first AUG in an appropriate Kozak context. Thus, for approximately 90% of mRNA transcripts, the 5′-most AUG initiates protein synthesis (Kozak 1991). In order to develop predictive algorithms for the CUG initiation codon, we asked whether ribosomes similarly scanned for the CUG/leucine start. We took advantage of the fact that a heat-stable hairpin in the mRNA blocks 40S ribosomal scanning (Kozak 1986, 1989a). We designed a hairpin based on the one Kozak used to trap scanning 40S ribosomes (Kozak 1989b). It extends Kozak's original sequence in order to maintain stability at 37 °C, and includes a bulge to prevent the longer hairpin from targeting the mRNA as a potential substrate for RNA interference. We placed the hairpin 42 nucleotides upstream of the initiation codon. To control for nonspecific effects, such as RNA stability, we also included GFP under the control of an IRES element downstream of the peptide-coding sequence.

**Figure 5:**
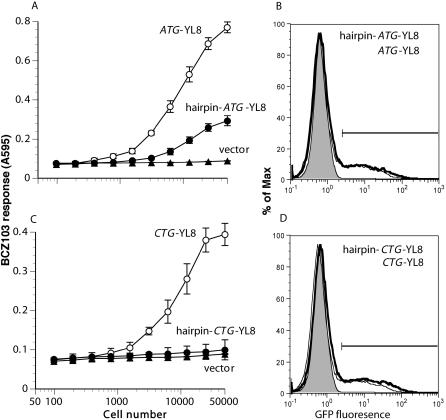
The CUG Start Is Blocked by a Heat-Stable Hairpin in the 5′ UTR COS-7 cells were transfected with cDNA encoding K^b^ and the indicated constructs. (A and C) The cells were titrated and peptide expression was tested with BCZ103 T cells. (B and D) GFP expression in the transfected cells was assayed by fluorescence-activated cell sorting. GFP fluorescence (shaded histograms) is not observed in untransfected cells (or in cells transfected with a vector not encoding GFP [unpublished data]).

We first transfected COS-7 cells with constructs encoding the ATG-initiated MYL8 peptide and another encoding MYL8 downstream of the heat-stable hairpin. We titrated the transfected cells and assayed the T cell response to the peptides presented on the cell surface. As expected, the presence of the hairpin inhibited MYL8 expression (Figure 5A). This effect was not due to the hairpin destabilizing the mRNA, because GFP expression as measured by flow cytometry of the same cells was not decreased by the presence of the hairpin (Figure 5B). Next, we performed the same experiment with COS-7 cells transfected with the CTG-initiated peptide with and without an upstream hairpin sequence and found that the hairpin inhibited translation of LYL8 as well (Figure 5C). Again, the level of GFP expression was similar in the cells transfected with constructs with or without the hairpin (Figure 5D). Similar inhibition of both ATG- and CTG-initiated translation was also observed with a second set of constructs in which the hairpin was placed 68 nucleotides upstream of the initiation codon, making it less likely that the hairpin interfered with a potential IRES-like ribosomal landing pad (unpublished data). Both conventional, ATG-mediated and cryptic, CTG-mediated translation events were disrupted by upstream hairpins, suggesting that both require ribosomal scanning in the 5′-to-3′ direction.

### A Set of Ribosomes Is Scanning Specifically for the CUG/Leucine Start

We next asked whether ribosomes responsible for the CUG/leucine start were scanning specifically for CUG initiation codons, or whether they were able to start at conventional AUG initiation codons as well. To address this question, we placed “decoy” ATG and CTG codons upstream of and out of frame with the CTG codon initiating expression of the peptide, and asked whether their presence affected peptide translation.

**Figure 6:**
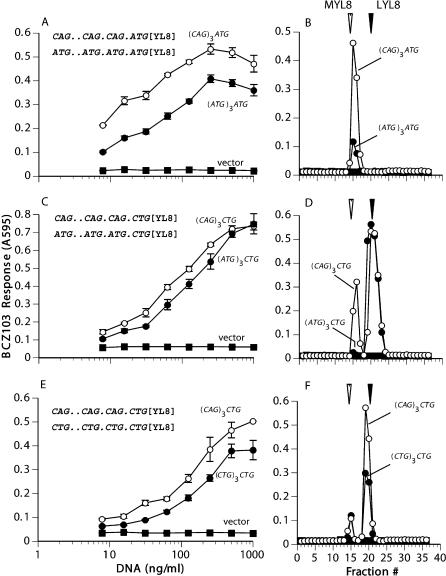
Ribosomes Are Scanning Specifically for the CUG/Leucine Start (A, C, and E) Lmtk^–^ cells were transfected with the indicated constructs and K^b^ cDNA. After 2 d they were tested for MYL8/K^b^ or LYL8/K^b^ expression using the BCZ103 T cell hybridoma. Error bars represent the standard deviation of three replicate wells. (ATG)_3_ATG (solid circles, A) denotes the ATG-initiated peptide preceded by three ATGs upstream of and out of frame with the peptide; (CAG)_3_ATG (open circles, A) is the identical DNA construct but the upstream ATGs were replaced with CAG. (ATG)_3_CTG (solid circles, C) denotes the CTG-initiated peptide preceded by three ATGs upstream of and out of frame with the peptide; (CAG)_3_CTG (open circles, C) is the identical DNA construct but the upstream ATGs were replaced with CAG. (CTG)_3_CTG (solid circles, E) denotes the CTG-initiated peptide preceded by three CTGs upstream of and out of frame with the peptide; (CAG)_3_CTG (open circles, E) is the identical DNA construct but the upstream CTGs were replaced with CAG. (B, D, and F) Extracts from COS-7 cells transfected with cDNA encoding K^b^ and the indicated constructs were separated by RP-HPLC. Fractions were tested for BCZ103 T cell-stimulating activity with K^b+^B7.2^+^ L cells as APCs. Arrows indicate the peak elution positions of the MYL8 and the LYL8 peptides. Points on graphs correspond to those in (A), (C), and (E).

As a positive control, we transfected cells with a construct encoding the ATG-initiated MYL8 and another encoding the same MYL8 peptide but with three ATGs upstream of and out of frame with the peptide (ATG)_3_ATG. The control constructs had CAGs instead of ATGs, because the CAG codon does not possess initiation activity. As expected, the presence of upstream out-of-frame ATGs dramatically reduced ATG-initiated MYL8 peptide expression. The reduction in MYL8 peptide was seen both when the transfected cells were used directly to stimulate T cells and when peptides from the transfected cells were extracted, separated by HPLC, and then assayed with T cells (Figure 6A and 6B). This result is in complete agreement with the scanning model of translation initiation. The marked reduction in peptide expression occurs because ribosomes initiate translation at the first AUG and traverse the downstream peptide-coding region in the wrong reading frame; the ribosomes that missed the first AUG would start at the second or the third AUG and also traverse the peptide coding region in the wrong reading frames (Bullock and Eisenlohr 1996; Bullock et al. 1997).

We then transfected cells with a construct encoding (CTG)YL8 and another encoding (CTG)YL8 with three ATGs upstream of and out of frame with the peptide (ATG)_3_CTG. When the transfected cells were used directly to stimulate T cells, the upstream ATGs had little effect (Figure 6C). However, HPLC analysis of the peptides produced in transfected cells revealed that the upstream ATGs virtually abolished expression of the MYL8 peptide, whereas LYL8 expression was not affected (Figure 6D). Thus, upstream out-of-frame ATG initiation codons inhibited the conventional “wobble” CUG/methionine start, but not the CUG/leucine start.

Finally, we transfected COS-7 cells with constructs encoding (CTG)YL8 with three CTGs upstream of and out of frame with the peptide (CTG)_3_CTG. When the transfected cells were used directly to stimulate T cells, we saw a small but consistent inhibition (Figure 6E). Remarkably, HPLC analysis of the transfected cell extracts showed that only LYL8 expression was decreased without any change in the expression of the MYL8 peptide (Figure 6F). The observation that upstream ATGs inhibit the wobble start but not the leucine start, and that upstream CTGs inhibit the leucine start but not the wobble start, suggests that a separate set of ribosomes is scanning for the CUG/leucine start, distinct from the ribosomes used for the methionine start.

Note, however, that the upstream CUGs, despite an “Excellent Kozak” context, inhibited the leucine start weakly. This effect contrasts with the inhibition caused by the upstream AUGs on the AUG/methionine or the CUG/methionine starts and suggests that other features are required for an efficient CUG/leucine start. Interestingly, one form of the ASCT2 amino acid transporter is initiated with multiple CUG and GUG codons in close proximity (Tailor et al. 2001). This redundancy may be required if any given non-AUG codon is used inefficiently. Furthermore, many mRNAs with CUG starts have GC-rich regions immediately downstream from the initiation codon and have been hypothesized to form hairpins that cause the ribosome to pause long enough to recognize the CUG codon (Kozak 1990).

### The Leucine Start Is Enhanced in the Presence of Phosphorylated Eukaryotic Translation Initiation Factor 2α

Finally, we were interested to know whether the leucine start requires eukaryotic translation initiation factor 2 (eIF2), which is responsible for loading the RNA_i_^Met^ onto the 40S ribosome. Cells target eIF2 by phosphorylating its α subunit (eIF2α) to inhibit protein synthesis in response to a number of stress signals, including viral infection, starvation, and the accumulation of unfolded proteins. Ribosomes release eIF2-guanosine diphosphate (GDP) after the AUG initiation codon is reached, and GDP is exchanged for guanosine triphosphate (GTP) with the assistance of another protein, eIF2B, before eIF2 can be used for another round of translation initiation. When eIF2α is phosphorylated, it binds eIF2B with unusually high affinity and thus prevents subsequent nucleotide exchange. Because eIF2B is limiting in the cell, phosphorylation of only a fraction of eIF2α can substantially inhibit translation globally (Hershey and Merrick 2000).

To approach this question, we transfected HeLa cells with (CTG)YL8 or (ATG)YL8 constructs. We then assayed peptide expression in cells that had or had not been treated to induce phosphorylation of eIF2α. It was a challenge to induce phosphorylation of eIF2α for long enough to see an effect on peptide expression without causing substantial toxicity. Furthermore, we could not disrupt peptide/MHC assembly in the endoplasmic reticulum, a requirement that ruled out standard reagents such as dithiothreitol, thapsigargin, and tunicamycin. We also did not want to inhibit peptide elongation, which ruled out amino acid starvation.

**Figure 7:**
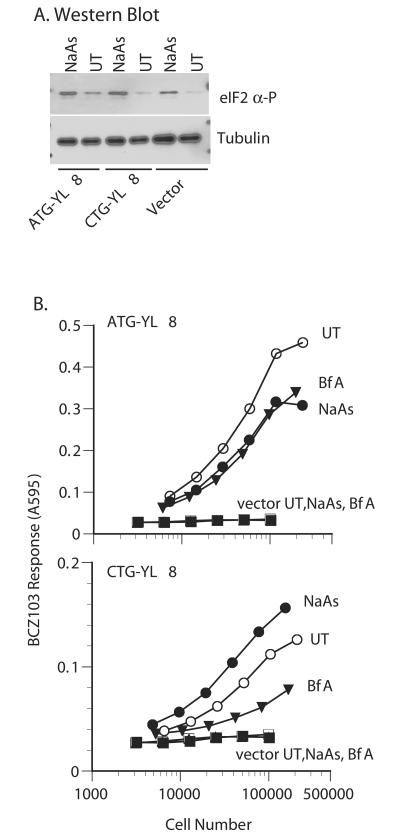
The Leucine Start Is Enhanced in the Presence of Phosphorylated eIF2α HeLa cells transfected with cDNA encoding K^b^ together with cDNA encoding either the ATG- or CTG- initiated peptides were treated for 4 h with 50 μM NaAs, with brefeldin A (BfA), or left untreated (UT). (A) Transfected cells treated with NaAs or without (UT) were lysed and tested for phosphorylation of eIF2α by Western blot and for tubulin as a loading control. (B) The transfected cells were titrated and tested for their ability to stimulate BCZ103 T cells.

The optimal treatment for our purposes was sodium arsenite (NaAs). Arsenite reacts with sulfhydryl groups and causes phosphorylation of eIF2α presumably by inducing an unfolded protein response, although the precise mechanism remains unknown (Brostrom and Brostrom 1998). We transfected HeLa cells with DNA encoding the CTG and ATG-initiated peptides. After 12 h, we treated the cells with 50 μM NaAs for 4 h before assaying them for expression of eIF2α and peptides. Western blot analysis confirmed that the amount of phosphorylated eIF2α in NaAs-treated cells was enhanced (Figure 7A). As a control, the amount of tubulin in the cells remained unchanged.

We assayed the HeLa cells for peptide expression using the BCZ103 T cell hybridoma (Figure 7B). We found that expression of the ATG-initiated MYL8 peptide was reduced by NaAs, as expected. The reduction was dramatic because the effect of NaAs was equivalent to the effect of brefeldin A, which stops protein transport from the endoplasmic reticulum to the Golgi and thus prevents any new peptides from trafficking to the cell surface. Surprisingly, expression of the CTG-initiated LYL8 peptide increased upon NaAs treatment, although it too was inhibited by brefeldin A treatment. This result was consistent in six independent experiments. The difference in ATG versus CTG initiation is not likely due to a unique effect of NaAs that somehow stabilizes LYL8 and not MYL8, because an alternative method of inducing phosphorylation of eIF2α using β-interferon and polyIC (Savinova and Jagus 1997; Kaufman 2000), gave similar reproducible results (unpublished data). We conclude that, in contrast to initiation at conventional AUG codons, initiation at the CUG/leucine codon was eIF2α-independent.

The effect of eIF2α phosphorylation on the leucine start strikingly mirrors the effect of eIF2α phosphorylation on proteins whose synthesis is directed by the CPV-IRES, which does not require eIF2 (Wilson et al. 2000). Intriguingly, eIF2-independence of the CUG start is consistent with the observation of Donahue et al. (1988) that mutations in a Zn(II) finger domain of eIF2β permit initiation at a UUG codon. The data implicates the nucleic acid-binding function of eIF2β in start-site selection (Donahue et al. 1988; Huang et al. 1997). Identification of the factors required for the CUG start will illuminate not only leucine initiation, but also the role of these components in the methionine start.

The CPV-IRESs are to date the only known sequences that allow eIF2-independent initiation in eukaryotic cells. Viruses employ a host of creative strategies to prevent phosphorylation of eIF2α (Kaufman 2000). Initiation at non-AUG codons, which is relatively common in viral transcripts, may provide a way to continue translation despite the lack of eIF2. Similarly, despite general translational inhibition during viral infection, cells need to continue generating antigenic peptides to flag down T cells. In addition to viral proteins and antigenic peptides, a number of regulatory cellular proteins have non-AUG initiation codons. For example, c-Myc has two distinct isoforms, the longer of which is initiated with CUG and may inhibit proliferation, as it is absent in a number of tumor-derived cell lines. Intriguingly, synthesis of the CUG-initiated form increases when cells reach high density, specifically when methionine is limiting (Hann et al. 1988; Hann et al. 1992). It should be interesting to test whether this and other CUG-initiated proteins have a leucine start. In addition, whether other non-AUG initiation codons such as GUG or ACG are decoded in a manner similar to the CUG codon described here remains to be determined.

In summary, we found that when CUG acts as an alternate initiation codon, it can be decoded as leucine as well as methionine. The leucine start does not depend on mRNA structure or sequence, but its efficiency can be enhanced by the Kozak context. A set of ribosomes is scanning 5′ to 3′ specifically for the CUG initiation codon. While the methionine start is inhibited when cells are treated with NaAs, the leucine start is enhanced, suggesting that leucine initiation is independent of eIF2. This novel translation initiation mechanism provides cells not only antigenic peptides but also a potential tool for translational control.

## Materials and Methods

### 

#### Cell lines

Lmtk^–^, COS-7, K^b+^B7.2^+^ L, D^b+^B7.2^+^ L, BCZ103, 30NX/B10Z, and DBFZ cells have been described (Mendoza et al. 1997; Malarkannan et al. 1999). Cell lines were maintained at 37 °C with 5% CO_2_ in RPMI 1640 with 10% fetal bovine serum, 1 mM sodium pyruvate, 50 μM β-mercaptoethanol, 0.3 mg/ml glutamine, 100 U/ml penicillin, and 100 μg/ml streptomycin (normal medium). HeLa cells were obtained from ATCC (#CCL-2; Manassas, Virginia, United States), and cultured in Eagle's minimal essential medium modified with Earle's balanced salt solution, nonessential amino acids, 2 mM L-glutamine, 1 mM sodium pyruvate, 1500 mg/l sodium bicarbonate (ATCC #30–2003), 10% fetal bovine serum, and 10 μg/ml ciprofloxacin.

#### Plasmid construct sequences

Sequences for constructs depicted in Figure 1 are as follows. CTG-YL8 in the BstXI/XbaI sites of the pcDNA1 vector (Invitrogen, Carlsbad, California, United States), 5′-TGTGTAGCTGACCTTCAACTACCGGAATCTATAGCTAG-3′; CTG-YL8 in the EcoRI/BamHI sites of the pIRES2-eGFP vector (Clontech, Palo Alto, California, United States), 5′-AATTAGACGAAGGTCTAGCTGACCTTCAACTACCGTAACCTGTAGATC-3′; CTG-YL8 in the HindIII/BamHI sites of the pcDNA1 vector with GFP in the EcoRI site, 5′-AGCTAGCTGACCTTCAACTACCGGAATCTATAGATCGATC-3′; ATG-YL8 in the EcoRI/BamHI sites of the pIRES2-eGFP vector, 5′-AATTAGACGAAGGTCTAGATGACCTTCAACTACCGTAACCTGTAGATC-3′; CTG-M9 in the BstXI/XbaI sites of the pcDNA1 vector, 5′-TGTGTAGCTGAGCAACGAGAACATGGAGACCATGTAGTGCACTAG-3′; and ATG-M9 in the BstXI/XbaI sites of the pcDNA1 vector, 5′-TGTGTAGATGAGCAACGAGAACATGGAGACCATGTAGTGCACTAG-3′.

Sequences for constructs depicted in Figure 2 are as follows. R0 in the BstXI/XbaI sites of the pcDNA1 vector, 5′-TGTGTAGATGAGCAGCGTCGTCGGCGTTTGGTACCTCTAGCTGACCTTCAACTACCGGAATCTCTAG-3′; and R1 in the BstXI/XbaI sites of the pcDNA1 vector, 5′-TGTGTAGATGGAGCAGCGTCGTCGGCGTTTGGNACCTCTAGCTGACCTTCAACTACCGGAATCTCTAG-3′.

Sequences for constructs depicted in Figure 3 are as follows. CTG-initiated peptide in the BstXI/XbaI sites of the pcDNA1 vector, 5′-TGTGTAGNCCNCCCTGNCCAGTGTTGTTGAATTCTCCAGCCTCTAG-3′; and CCC-initiated peptide in the BstXI/XbaI sites of the pcDNA1 vector, 5′-TGTGTAGNCCNCCCCCNCCAGTGTTGTTGAATTCTCCAGCCTCTAG-3′.

Sequences for constructs depicted in Figure 4 are as follows. “Excellent Kozak” CTG-YL8 in the BstXI/XbaI sites of the pcDNA1 vector, 5′-TGTGTAGTCGACCCTGACCTTCAACTACCGGAATCTCTAG-3′; and “Poor Kozak” CTG-YL8 in the BstXI/XbaI sites of the pcDNA1 vector, 5′-TGTGTAGGCGTCCCTGACCTTCAACTACCGGAATCTCTAG-3′.

Sequences for constructs depicted in Figure 5 are as follows. CTG-YL8 or ATG-YL8 in the pIRES2-eGFP vector, as in Figure 1 hairpin CTG-YL8 or ATG-YL8 in the pIRES2-eGFP vector (sequence from 5′ cap to start of peptide, whose sequence is as in Figure 1): 5′-GCTAGCGCTACCGGACTCAGATCGTGTCCGGATTTGGGGCGCGTGGTGGCGGCTTTTCGCGCGCGCGACGCGTCGCGCGCGCGTTTTGCCGCCACCACGCGCCCCTTTAGTACTTGAGCTCAAGCTTCGAATTAGACGAAGGTCTAG-3′.

Sequences for constructs depicted in Figure 6A and 6B are as follows. CAG_3_ATG-YL8 in the BstXI/XbaI sites of the pcDNA1 vector, 5′-TGTGAACCCAGGGTCGACCCAGGACCCAGGTAGTCGACCATGACCTTCAACTACCGGAATCTCTAG-3′; and ATG_3_ATG-YL8 in the BstXI/XbaI sites of the pcDNA1 vector, 5′-TGTGAACCATGGGTCGACCATGGACCATGGTAGTCGACCATGACCTTCAACTACCGGAATCTCTAG-3′.

Sequences for constructs depicted in Figure 6C and 6D are as follows. CAG_3_CTG-YL8 in the BstXI/XbaI sites of the pcDNA1 vector, 5′-TGTGAACCCAGGGTCGACCCAGGACCCAGGTAGTCGACCCTGACCTTCAACTACCGGAATCTCTAG-3′; and ATG_3_CTG-YL8 in the BstXI/XbaI sites of the pcDNA1 vector, 5′-TGTGAACCATGGGTCGACCATGCACCATGGTAGTCGACCCTGACCTTCAACTACCGGAATCTCTAG-3′.

Sequences for constructs depicted in Figure 6E are as follows. CAG_3_CTG-YL8 in the HindIII/XbaI sites of the pcDNA1 vector mutated to alter the two CTGs in the 5′ UTR to CAG, 5′-AGCTACAAACAGGATTACAAACAGGATTACAAACAGGATTACAAACAGGATTACAAACAGGATTACAAACAGGATTACAAACAGGATTACAAACAGGATTACAAACAGGATTTAGAAACTGACCTTCAACTACCGGAATCTCTAG-3′; and CTG_3_CTG-YL8 in the HindIII/XbaI sites of the pcDNA1 vector mutated to alter the two CTGs in the 5′ UTR to CAG, 5′-AGCTACAAACAGGATTACAAACAGGATTACAAACAGGATTACAAACAGGATTACAAACAGGATTACAAACAGGATTACAAACTGGATTACAAACTGGATTACAAACTGGATTTAGAAACTGACCTTCAACTACCGGAATCTCTAG-3′

Sequences for constructs depicted in Figure 6F are as follows. CAG_3_CTG-YL8 in the HindIII/XbaI sites of the pcDNA1 vector, 5′-AGCTACAAACAGGNTTACAAACAGGATTACAAACAGGATTACAAACAGGATTACAAACAGGATTACAAACAGGATTACAAACAGGATTACAAACAGGATTACAAACAGGATTTAGAAACTGACCTTCAACTACCGGAATCTCTAG-3′; and CTG_3_CTG-YL8 in the HindIII/XbaI sites of the pcDNA1 vector, 5′-AGCTACAAACAGGATTACAAACAGGATTACAAACAGGATTACAAACAGGATTACAAACAGGATTACAAACAGGATTACAAACTGGATTACAAACTGGATTACAAACTGGATTTAGAAACTGACCTTCAACTACCGGAATCTCTAG-3′.

Sequences for constructs depicted in Figure 7 are as follows. CTG-YL8 in the in the BstXI/XbaI sites of the pcDNA1 vector, 5′-TGTGTAGCTGACCTTCAACTACCGGAATCTGTAGCTAG-3′; ATG-YL8 in the in the BstXI/XbaI sites of the pcDNA1 vector, 5′-TGTGAAAAACAGGCCAAACAGGCCAAACAGGAAGATGACCTTCAACTACCGGAATCTCTAG-3′.

#### Transfections

The DEAE-dextran transfection method was used in Figures 2B and 2C, 3, 4A, 6A, 6C, and 6E. It has been described previously (Serwold et al. 2001). Briefly, Lmtk^–^ or COS-7 cells were transfected in 96-well plates (1 × 10^4^ cells per well) with the indicated concentration of plasmid DNA, 0.1 mg/ml DEAE-dextran, 0.1 mM chloroquine, 10 ng/ml MHC class I cDNA, 5 ng/ml B7.2 cDNA, and 10% NuSerum (Collaborative Biomedical Products, Becton Dickinson, Bedford, Massachusetts, United States) in RPMI 1640. After 90 min, the cells were shocked with 10% DMSO in PBS for 2 min. After 2 d incubation in normal medium, T cells (1 × 10^5^ cells per well) were added to assay peptide expression. GeneJuice (Novagen, Madison, Wisconsin, United States) transfection reagent was used in Figures 1, 2D, 4B, 5, 6B, 6D, 6F, and 7. It was used according to the manufacturer's instructions, except that we used 8 ml of medium, 8 μl of GeneJuice, 1.33 μg of MHC class I DNA, and 1.33 μg of peptide DNA per 10-cm dish. Peptide expression in the transfected cells was assayed after 2 d by titrating intact cells or after fractionating the cell extracts by HPLC.

#### T cell assay

The T cell assay has been described previously (Sanderson and Shastri 1994). Briefly, Lac Z-inducible T hybridomas (1 × 10^5^ cells) were incubated with APCs in 96-well plates for 6–24 h. The response, accumulation of intracellular β-galactosidase, was measured with the substrate chlorophenol red-β-D-galactopyranoside (CPRG). The product was measured with a 96-well plate reader at 595 nm and 655 nm as the reference wavelength.

#### HPLC

The HPLC assay has been described previously (Serwold et al. 2001). Briefly, 2 d after transfection, cells were lysed in 10% formic acid, and the lysate was passed through a 10 kDa cutoff filter (Millipore, Bedford, Massachusetts, United States). The <10 kDa fraction was injected onto a 2.1 × 250 mm C18 column (Vydac, Hesperia, California, United States) and separated by RP-HPLC, with 0.1% TFA in water as the polar buffer and 0.1% TFA in acetonitrile as the nonpolar buffer. Three-drop fractions (except analysis of xM9, with five-drop fractions) were collected in 96-well plates, dried in a vacuum centrifuge, and analyzed by the addition of APCs (5 × 10^4^ per well) and T cell hybridomas (1 × 10^5^ cells per well).

#### eIF2α phosphorylation

Cells were transfected with GeneJuice (Novagen) as described above, but the transfection medium was left on for only 4 h before the cells were lifted and split into multiple dishes with fresh medium. Cells were allowed to rest for 12 h before sodium arsenite treatment. They were treated with 50 μM NaAs (Sigma, St. Louis, Missouri, United States), 1× GolgiPlug containing brefeldin A (PharMingen, San Diego, California, United States), or left untreated for 4 h. The cells were then lifted and counted. For the T cell assay, they were titrated in a 96-well plate. The assay was as described above, except that 1× GolgiPlug was added in order to “freeze” the cells in their state at the end of treatment. For the Western blot, they were incubated on ice for 5–10 min in lysis buffer (20 mM HEPES [pH 7.5], 150 mM NaCl, 1% Triton X-100, 10% glycerol, 1 mM EDTA, 10 mM tetrasodium pyrophosphate, 100 mM NaF, 17.5 mM β-glycerophosphate, 0.4 U/ml aprotinin, 10 μg/ml leupeptin, 1 mM PMSF, 0.1 mM pepstatin A, and complete protease inhibitor cocktail [Roche, Basel, Switzerland]). The lysate was spun for 15 min at 4 °C, and the supernatant was transferred to a tube containing an equal volume of 2× SDS-PAGE sample buffer (100 mM Tris-HCl [pH 6.8], 20% glycerol, 4% SDS, bromophenol blue, and 5% β-mercaptoethanol). The sample was then heated in water just off the boil for 5 min, separated on a 10% SDS-PAGE gel, and transferred to a nitrocellulose membrane. The membrane was blocked for 1 h at room temperature in TBS–0.1% Tween 20–5% bovine serum albumin (TBS, 0.02 M Tris-HCl [pH 7.6] with 0.137 M NaCl), incubated for 1 h with primary antibody (#44–728, at a 1:2000 dilution; Biosource, Camarillo, California, United States) in TBS–0.1% Tween 20–5% bovine serum albumin, washed four times for 5 min with TBS–0.1% Tween 20, incubated for 40 min with secondary antibody (anti-rabbit-HRP #NA934V, at a 1:30,000 dilution; Amersham, Little Chalfont, United Kingdom), washed four times for 5 min with TBS–0.1% Tween 20, incubated for 5 min in substrate (SuperSignal West Femto Maximum Sensitivity Substrate, #34095; Pierce Biotechnology, Rockford, Illinois, United States), and exposed to film. An antibody to α-tubulin (#sc-5546; Santa Cruz Biotechnology, Santa Cruz, California, United States) was used as a loading control.

## Abbreviations

APC: antigen-presenting cell
CPV-IRES: cricket paralysis virus-like internal ribosome entry site
CTL: cytotoxic T cell
eIF2: eukaryotic translation initiation factor 2
eIF2α: eIF2 α subunit
GDP: guanosine diphosphate
GFP: green fluorescent protein
GTP: guanosine triphosphate
HPLC: high performance liquid chromatography
IRES: internal ribosome entry site
MHC: major histocompatibility complex
NaAs: sodium arsenite
RNA_i_^Met^: methionyl initiator RNA
RP-HPLC: reverse-phase HPLC
UTR: untranslated region
